# Retraction statement: Shao, L., Zhang, X. and Yao, Q. (2020), The F‐box protein FBXO11 restrains hepatocellular carcinoma stemness via promotion of ubiquitin‐mediated degradation of Snail. FEBS Open Bio, 10: 1810–1820.

**DOI:** 10.1002/2211-5463.13623

**Published:** 2023-05-23

## Abstract

https://doi.org/10.1002/2211-5463.12933

The above article, published online on 13 July 2020 in Wiley Online Library (wileyonlinelibrary.com), has been retracted by agreement between the between the authors, the Editor‐in‐Chief, FEBS Press, and John Wiley & Sons Ltd. The retraction has been agreed following an investigation into concerns raised by a third party, which revealed inappropriate duplications between this and articles either previously published or published in the same month [1–3]. Thus, the editors consider the conclusions of this manuscript substantially compromised.

Reference

1 Wu H, He Y, Chen H, Liu Y, Wei B, Chen G, Lin H and Lin H LncRNA THOR increases osteosarcoma cell stemness and migration by enhancing SOX9 mRNA stability. doi: 10.1002/2211‐5463.12620

2 Chen J, Wang P, Cai R, Peng H, Zhang C and Zhang M SLC34A2 promotes neuroblastoma cell stemness via enhancement of miR‐25/Gsk3β‐mediated activation of Wnt/β‐catenin signaling. doi: 10.1002/2211‐5463.12594

3 (2020) Long Non‐Coding RNA THOR Enhances the Stem Cell‐Like Traits of Triple‐Negative Breast Cancer Cells Through Activating β‐Catenin Signaling. *Med Sci Monit* **26**, e923507.doi: 10.12659/MSM.923507

https://doi.org/10.1002/2211-5463.12933

The above article, published online on 13 July 2020 in Wiley Online Library (wileyonlinelibrary.com), has been retracted by agreement between the between the authors, the Editor‐in‐Chief, FEBS Press, and John Wiley & Sons Ltd. The retraction has been agreed following an investigation into concerns raised by a third party, which revealed inappropriate duplications between this and articles either previously published or published in the same month [1–3]. Thus, the editors consider the conclusions of this manuscript substantially compromised.

**Reference**

1 Wu H, He Y, Chen H, Liu Y, Wei B, Chen G, Lin H and Lin H LncRNA THOR increases osteosarcoma cell stemness and migration by enhancing SOX9 mRNA stability. doi: 10.1002/2211‐5463.12620

2 Chen J, Wang P, Cai R, Peng H, Zhang C and Zhang M SLC34A2 promotes neuroblastoma cell stemness via enhancement of miR‐25/Gsk3β‐mediated activation of Wnt/β‐catenin signaling. doi: 10.1002/2211‐5463.12594

3 (2020) Long Non‐Coding RNA THOR Enhances the Stem Cell‐Like Traits of Triple‐Negative Breast Cancer Cells Through Activating β‐Catenin Signaling. *Med Sci Monit* **26**, e923507.doi: 10.12659/MSM.923507

